# The Balanced Two-String Technique for Sulcus Intraocular Lens Implantation in the Absence of Capsular Support

**DOI:** 10.1155/2015/153963

**Published:** 2015-02-02

**Authors:** Hesham A. Ibrahim, Heba Nabil Sabry

**Affiliations:** Department of Ophthalmology, Faculty of Medicine, Alexandria University, Raml Station, Alexandria, Egypt

## Abstract

*Purpose*. To describe and explore an alternative approach for sulcus intraocular lens (IOL) implantation in the absence of capsular support. *Methods*. The commonly available one-piece poly(methyl methacrylate) (PMMA) lens is stabilized in the sulcus by two intraocular horizontal strings of 10/0 polypropylene suture passed through the lens dialing holes in opposite directions to achieve a mechanical balance. The horizontal strings of 10/0 polypropylene work as a rail track for the IOL optics, allowing some side to side lens adjustment even following wound closure. The stability of the IOL was tested in vitro. Six aphakic patients underwent in-sulcus IOL secondary implantation using the balanced two-string technique. Patients were followed up for a minimum of six months. Best spectacle corrected vision was assessed. Lens centration and lens tilt were measured by anterior segment optical coherence tomography (AS-OCT). *Results*. All patients had successful lens insertion. Best spectacle corrected visual acuity (BSCVA) improved in all patients. Lens decentration ranged between 0.21 mm and 0.9 mm (average 0.53 mm). Lens tilt ranged between 1.2° and 2.8° (average 2.17°). *Conclusion*. The mechanically balanced two-string technique is an alternative option for sulcus IOL implantation in absence of capsular support, allowing lens centration adjustment with no additional risks.

## 1. Introduction

In the absence of capsular support intraocular lens (IOL) implantation behind the iris plane is still possible with various scleral fixation techniques [1, 2]. Globe collapse, lens decentration, lens tilt, and sutures related complications are known encountered difficulties with sclera fixated IOL (SF-IOL) [3–5]. Although more points of fixation allow greater stability with less risk of decentration and tilt [6], there are greater risk of complications from multiple sutures passing through the sclera and the uvea [7]. The IOL is usually fixated to the sclera via their haptics. Stability of haptic fixation was questionable and required special lens designs, haptic modifications, or adopting a technique that avoids the use of sutures for haptic fixation [6–11].

In this study, the widely available one-piece PMMA lens was stabilized in the sulcus by two 10/0 polypropylene horizontal strings. These strings were passed in a mechanically balanced way through the lens dialing holes. The technique addresses lens decentration, tilt, and sutures related complications.

## 2. Methods

**Figure 1:**
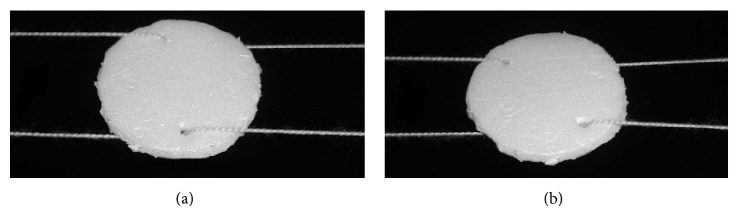
In vitro experiment on a cardboard disc showing disc resistance to tilt even with disc rotation.

The efficacy of the two mechanically balanced strings in stabilizing an IOL was experimented on a cardboard disc with two opposite peripheral holes mimicking the dialing holes of a PMMA IOL (Figure 1). A similar experiment was repeated in vitro with a PMMA lens suspended by two mechanically balanced strings of a 10/0 polypropylene suture through its dialing holes.

## 3. Surgical Technique

### 3.1. Surgical Steps (Figure 2)

**Figure 2:**
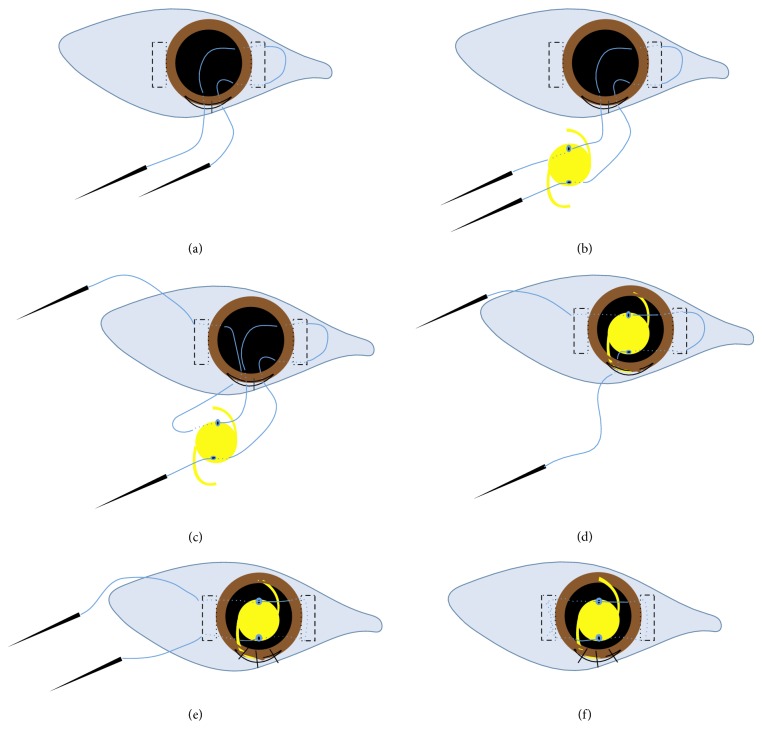
Diagrammatic illustration of the two balanced strings SF-IOL technique.

Temporal and nasal conjunctival periotomies are fashioned from 2 to 4 o'clock and from 10 to 8 o'clock. Two parallel 1/3 partial-thickness scleral grooves 5-6 mm long are made 2 mm from the limbus (at 3 and 9 o'clock). Two rectangular scleral pockets are dissected from each scleral groove towards the cornea by a crescent knife till they reach the limbus at 3 and 9 o'clock. A 7 mm superior corneal incision is fashioned and sutured to become water tight. The straight needles of a double-armed 10-0 polypropylene suture (Alcon STJ-6) are introduced into the eye by piercing the scleral bed at the upper and the lower extremities of the rectangular scleral pocket 2 mm from the limbus. The needles are directed in the iris plane to appear in the pupil. The needles are retrieved outside the globe from the corneal incision guided out by passing into the hollow tip of a 25-gauge needle inserted through the corneal incision. The lower and the upper needles are retrieved to the left and the right of the corneal wound (Figure 2(a)). The lower end of the 10-0 polypropylene suture passes through the lower dialing hole of the one-piece PMMA lens (EyeKon medical Inc., USA) from the front to the back surface of the lens and the upper needle passes through the upper dialing hole from the back to the front (Figure 2(b)). A 25-gauge needle is inserted under the lower extremity of the opposite (exit site) scleral pocket 2 mm from the limbus to emerge in the pupil for the retrieval of the lower polypropylene needle. The latter is reintroduced into the eye from the corresponding side of the corneal incision and passes into the hollow tip of the preplaced 25-gauge needle. The lower polypropylene needle is withdrawn out from the lower end of the opposite scleral pocket (Figure 2(c)). A viscoelastic is injected. The corneal wound sutures are cut and the PMMA posterior chamber IOL is inserted through the main corneal incision into the sulcus (Figure 2(d)). The corneal wound is secured with three interrupted 10/0 silk sutures and the intraocular pressure is reformed. The needle of the upper polypropylene string is withdrawn out from the upper extremity of the exit site scleral pocket by passing through the hollow of a preplaced 25-gauge needle. The lower and upper 10/0 polypropylene strings are pulled tight from their exit site to bury the polypropylene loop at the entry site within the scleral pocket (Figure 2(e)). Side to side lens centration is checked and readjusted along the two 10/0 polypropylene strings.

The two ends of the 10/0 polypropylene suture are tightened and tied. The knot is secured to the scleral bed within the scleral pocket (Figure 2(f)). The viscoelastic is removed, and the conjunctival periotomies are sutured. 

**Figure 3:**
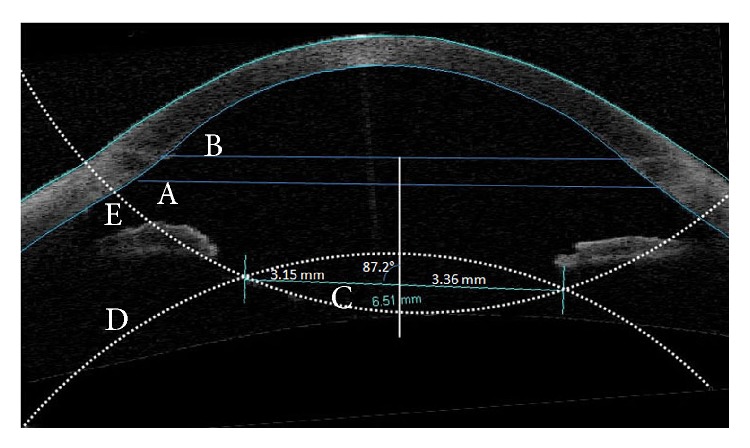
AS-OCT measuring lens tilt and decentration in a patient with irregular eccentric pupil. The perpendicular line posterior to the middle of the scleral spur line (A) and limbus line (B) transects the ICA-line (C). The anterior IOL curvature (D) and the posterior IOL curvature (E) were geometrically blotted to produce angles. ICA-line extends between these two angles. Lens decentration = difference between the left and right component of the transected ICA-line (3.36 mm − 3.15 mm = 0.21 mm). Lens tilt = the subtended angle deduced from 90° (90°−87.2° = 2.8°).

*Clinical Experience*. Six aphakic patients had SF-IOL by the two-balanced-string technique through the dialing holes (Table 1). The research adhered to the tenets of the Declaration of Helsinki and was conducted after the approval of Ethical Review Board (ERB) of Faculty of Medicine, Alexandria University. A written consent form was signed by patients. Preoperative evaluation included refraction, best spectacle corrected visual acuity (BSCVA), intraocular pressure (IOP) measurement, and fundus examination. The patient records were reviewed for intraoperative and postoperative complications and visual outcome. Patients who had preoperative noncorrectable BSCVA, corneal opacity, or retinal problems were excluded from this work. Lens tilt and decentration were assessed by Visante anterior segment OCT (Carl Zeiss Meditec, Dublin, CA, USA). A reference line was drawn between the scleral spur and/or the limbus on the opposite sides of the AS-OCT images. (Figure 3) The anterior and the posterior curvatures of the IOL surface were determined. The lens periphery on AS-OCT images was blotted geometrically by extending the IOL curvatures until they meet at right and left angles. A line was drawn between the lens curvature angles on both sides of the AS-OCT image to represent lens position. A perpendicular line to the scleral spur or the limbus reference lines was drawn from their middle to bisect the IOL curvature angles line (ICA-line). The difference in length between the resulting right and left segments of the ICA-line indicated the degree of lens decentration. The angle subtended by the perpendicular line to the ICA-line indicated the degree of IOL tilt.

## 4. Results (Table 1)

All patients had IOL implants as a secondary procedure. Age ranged between 22 y and 73 y (mean 51 y). Two patients had aphakia because of cataract surgery with loss of capsular support. One patient had preoperative subluxated cataractous lens and had primary intracapsular cataract extraction. One patient had lens removal with a cutter and irrigation aspiration due to zonular dehiscence. One patient had a sunset subluxated IOL implanted in the bag due to zonular dehiscence. One patient had traumatic cataract with lack of capsular support.

Preoperative BSCVA ranged between 20/80 and 6/60 mainly due to irregular astigmatism caused by sutures following the primary surgery or lens displacement. Two patients had limited intraoperative bleeding during the passage of sutures. Postoperative follow up ranged between 6 and 36 months. Postoperative BSCVA ranged between 20/20 and 20/80 measured at least four months following the removal of corneal sutures. Corneal sutures were removed after 2-3 months. All patients gained BSCVA. Lens decentration ranged between 0.21 mm and 0.9 mm (average 0.53 mm). Lens tilt ranged between 1.2° and 2.8° (average 2.17°). There were no other complications or a need for another intervention.

## 5. Discussion

Variable approaches for scleral fixation are described in literature with common documented difficulties: lens tilt, decentration, and sutures related complications [2, 4, 8, 12]. In most scleral fixation techniques lens haptics are fitted directly to the sclera. That is why once the haptics are fixated, lens readjustment can be quite difficult. In this work, the IOL is suspended in the ciliary sulcus through its optics by two horizontally placed strings passing through the lens dialing holes. The two mechanically balanced strings prevent lens tilting as proved by the in vitro experiment. Vertical and horizontal centration of the IOL is dependent in one plane on accurate symmetrical location of the suture entry sites, but it can be adjusted along the parallel sutures in the orthogonal plane, which is a potential advantage. The scleral entry and exit sites can be precisely determined because the direction of scleral penetration is always from the outside to the inside. The two polypropylene strings are in fact a single loop and require only one knot that can be sutured and secured adequately enough to the bed of the scleral pocket under proper tension. The polypropylene suture is placed isolated from the subconjunctival space within the scleral pocket to avoid producing a track for infection. In comparison to the standard use of scleral flaps for other SF-IOL techniques, a scleral pocket would not require suturing. Scleral collapse during IOL suturing is managed by frequently reforming the intraocular pressure, which is helped by the temporary corneal stitches. Using the commonly available one-piece PMMA lens is an advantage. No lens modification is required. There is also no need to keep special lens design in stock which can be difficult to keep in most ophthalmic theaters.

The main difficulties with this technique are that it requires an attentive skillful surgeon, who should pay utmost care for choosing the proper site and direction of needle insertion, guard against sutures entangling, and adjust string tension on a reformed globe. Too tight loop may lead to cheese wiring and too lax loop would cause lens sagging. This technique also requires a 7 mm wound for PMMA lens insertion and consequently wound sutures, which can induce astigmatism.

Visual results are comparable to other reported SF-IOL techniques [1–12]. The degree of lens tilt and decentration were comparable to in-the-bag IOL lens implantation [13]. AS-OCT was used in this study to measure IOL tilt and decentration. IOL periphery is usually obscured by the iris in AS-OCT images. The IOL surface curvature angles were determined by geometrically blotting IOL periphery along the well determined central IOL surface curvatures. The IOL surface curvatures angles may not match precisely the actual lens edges but would have a symmetrical relation to them on both sides of the AS-OCT images. Deduction of the right and the left segments from each other would provide a precise measurement for IOL decentration. The ICA-line was determined on the vertical and horizontal meridians and was correlated to a reference scleral spur line or limbus line. This measurement technique provides precise IOL plane-representing line suitable for different lens edge designs.

Suture break was reported in literature to occur long time after the surgery. The use of a more durable suture material like polytetrafluroethylen (Gore-Tex) or a thicker (9-0 instead of 10-0) polypropylene suture material would be a safer option and was suggested by some authors [12, 14].

This work sheds light on an alternative technique to implant one-piece PMMA IOL in the absence of capsular support that may be of value in certain surgical circumstances.

## Figures and Tables

**Table 1:** Pre- and postoperative results for patients who underwent the balanced two strings scleral fixation IOL.

Age	Causes of aphakia	Preoperative BSCVA	Postoperative BSCVA	Complications	Lens decentration	Lens tilt
35 y/M	Congenital lens subluxation	20/80	20/20	—	0.25 mm	1.7°
48 y/F	Sunset intrabagal IOL due to zonular dehiscence	20/80	20/30	—	0.75 mm	2°
65 y/F	Post-ICCE	20/80	20/40	—	0.9 mm	2.5°
73 y/F	Postphaco	20/200	20/40	Limited vitreous hemorrhage	0.25 mm	2.8°
62 y/F	Postphaco	20/120	20/30	Limited vitreous hemorrhage	0.45 mm	1.2°
22 y/M	Posttraumatic cataract	20/120	20/80		0.75 mm	2.8°
